# Small Toxic Molecule Detection and Elimination Using Molecularly Imprinted Polymers (MIPs)

**DOI:** 10.3390/bios15060393

**Published:** 2025-06-18

**Authors:** Min Seok Kang, Jin-Ho Lee, Ki Su Kim

**Affiliations:** 1 School of Chemical Engineering, Pusan National University, 2 Busandaehak-ro 63 Beon-gil, Geumjeong-gu, Busan 46241, Republic of Korea; 2 Department of Information Convergence Engineering, Pusan National University, 49 Busandaehak-ro, Yangsan 50612, Republic of Korea; 3 School of Biomedical Convergence Engineering, Pusan National University, 49 Busandaehak-ro, Yangsan 50612, Republic of Korea; 4 Research Institute of Convergence of Biomedical Science and Technology, Pusan National University of Yangsan Hospital, Yangsan 50612, Republic of Korea; 5 Department of Organic Material Science & Engineering, Pusan National University, 2 Busandaehak-ro 63 Beon-gil, Geumjeong-gu, Busan 46241, Republic of Korea; 6 Institute of Advanced Organic Materials, Pusan National University, 2 Busandaehak-ro 63 Beon-gil, Geumjeong-gu, Busan 46241, Republic of Korea

**Keywords:** molecular imprinted polymer, small toxic molecules, sensing, elimination

## Abstract

Molecularly imprinted polymers (MIPs) provide selective, robust, and cost-effective platforms for the detection and removal of small toxic molecules in environmental, food, and biomedical contexts. This review offers a comprehensive overview of recent advancements in MIP-based systems, emphasizing critical design factors such as template selection, functional monomers, polymerization methods, and binding kinetics. The impact of these parameters on improving sensitivity, selectivity, and reusability is thoroughly examined. Additionally, current advantages, limitations, and enduring challenges are addressed. By highlighting emerging strategies and interdisciplinary innovations, this work aims to guide the development of more efficient and sustainable technologies for small-molecule toxin detection and remediation.

## 1. Introduction

Small toxic molecules, including environmental pollutants [1,2], industrial chemicals [3,4,5], pharmaceutical residues [6], and naturally occurring toxins [7], pose significant threats to human health. These compounds, often present in trace amounts, can cause severe biological and environmental consequences, such as water and soil contamination, bioaccumulation, and adverse health effects in humans and animals [8]. The persistent nature and widespread distribution of these toxic molecules necessitate highly sensitive, selective, and efficient methods for their detection and removal to ensure environmental safety, food security, and public health [9].

Despite advances in analytical and remediation technologies, existing methods often fail to meet these demands in practical use [10]. Conventional detection techniques such as gas chromatography (GC) [11], high-performance liquid chromatography (HPLC) [12], and mass spectrometry (MS) provide high sensitivity and accuracy for detecting toxic molecules [13]. However, these techniques are typically expensive, labor-intensive, and require sophisticated instrumentation and trained personnel [14]. Similarly, conventional removal strategies, including activated carbon filtration [15], chemical degradation [16], and biological treatments [17], often suffer from limitations such as non-selectivity, secondary pollution, and inefficiency at low concentrations [18]. These limitations highlight the urgent need for alternative materials capable of providing both selective detection and efficient removal of small toxic compounds.

In this context, molecularly imprinted polymers (MIPs) have emerged as a promising alternative solution to these challenges [19]. These synthetic polymeric materials are engineered with specific recognition sites that enable selective binding of target molecules based on a “lock-and-key” mechanism [20,21]. In contrast to biological receptors such as antibodies, aptamers, or enzymes, MIPs exhibit greater stability under extreme environmental conditions, resistance to degradation, and reusability over multiple cycles [22,23,24]. These outstanding properties make them ideal for applications in environmental monitoring [25], industrial wastewater treatment [26], and medical diagnostics [27,28].

However, the successful implementation of MIPs heavily depends on rational design strategies tailored to the target analytes and intended application [29,30,31]. Their performance hinges on several interdependent design parameters, including the choice of template molecule, selection of functional monomers, and polymerization method [23,32]. Each of these factors contributes to the specificity, binding affinity, and structural integrity of the imprinted sites [33]. For example, the choice of template molecule or its structural fidelity plays a pivotal role in defining the specificity of the imprinted recognition sites, while the selection of functional monomers directly influences the binding affinity [26,34]. Moreover, the polymerization method, including bulk polymerization, precipitation polymerization, or surface imprinting, also significantly influences the morphology and binding efficiency of MIPs [35]. Optimizing binding kinetics and reversibility is essential for achieving rapid and reliable adsorption and desorption, while maintaining long-term material stability and recognition performance is critical for practical use [36].

Recent advances in material science and nanotechnology have further advanced the development of MIPs into next-generation platforms [37,38]. Innovations such as nanostructured MIPs, hybrid composite materials, and integration with micro- and nanosensors have significantly enhanced the sensitivity, miniaturization, and multifunctionality of MIP-based systems [39,40,41]. Such advancements open promising avenues for the simultaneous detection and elimination of toxic molecules in complex matrices, including wastewater [37], biological fluids [42], and food samples [43]. However, these advantages can only be fully realized through rational design strategies that optimize key parameters, such as monomer–template interactions, polymerization techniques, and binding kinetics, which are critical for achieving high performance in detection and elimination applications [44].

**Figure 1:**
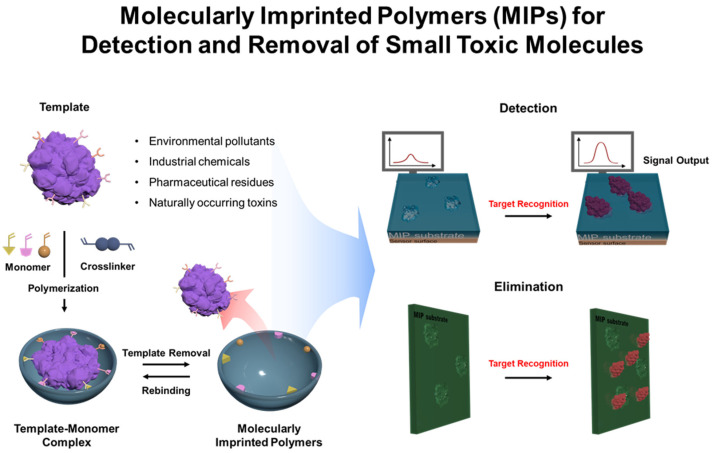
Schematic illustration of MIP-based detection and elimination of small toxic molecules using molecularly imprinted polymers.

In this review, we aim to provide a comprehensive overview of recent advancements in MIPs for the detection and elimination of small toxic molecules (Figure 1). We will explore key design considerations, including template selection, functional monomers, polymerization methods, and binding kinetics, and examine how these factors contribute to optimizing MIP performance. This analysis will highlight the potential of MIPs in overcoming challenges related to sensitivity, selectivity, and reusability, which are critical for effective detection and remediation of toxic substances. Additionally, we will discuss the strengths, limitations, and ongoing challenges associated with MIP-based approaches for future development. We hope this review will inspire interdisciplinary research, fostering the creation of more efficient and sustainable systems for toxin management in environmental and biomedical applications.

## 2. Rational Design Strategies for MIPs in the Detection and Elimination of Toxic Molecules

Molecularly imprinted polymers (MIPs) are synthetic materials with tailor-made recognition sites, designed to bind specific target molecules with high affinity and selectivity [45]. Their application in the detection and elimination of small toxic molecules has gained increasing attention due to their robustness, cost-effectiveness, and compatibility with various sensing and remediation platforms. The performance of MIPs is heavily dependent on their molecular design, which dictates the structural fidelity, binding site functionality, and overall selectivity of the polymer network [46]. Effective MIP design hinges on the careful optimization of several key parameters, including the functional monomer, cross-linker, template molecule, porogenic solvent, and polymerization technique. These variables must be finely tuned not only to enhance analytical performance but also to ensure practical functionality in complex environmental or biological matrices where toxic molecules are typically present at trace levels.

The core of MIP design lies in the selection of functional monomer, which interacts with the target molecule, known as the template, through specific intermolecular forces during the pre-polymerization phase. These interactions can be non-covalent (e.g., hydrogen bonding, ionic interactions, π–π stacking, hydrophobic effects), covalent, or semi-covalent [47,48]. For toxic small molecules, non-covalent imprinting is the most widely used strategy due to its simplicity and compatibility with a broad range of analytes. Among the most common functional monomers are methacrylic acid (MAA), which forms strong hydrogen bonds with polar or proton-donating templates; 4-vinylpyridine, known for its affinity toward acidic or electron-deficient molecules; and acrylamide, suitable for forming polar interactions [49,50,51]. The spatial orientation and electronic complementarity between the monomer and the toxic molecule are crucial in determining the quality of the imprinted cavity, which in turn governs the selectivity of the final polymer. Moreover, incorporating monomers with redox or fluorescent properties can enhance the functionality of MIPs for use in signal-transducing detection platforms [52].

The cross-linker is equally important, as it determines the rigidity and dimensional stability of the polymer matrix. It essentially “freezes” the three-dimensional configuration of the pre-polymerization complex after polymerization [53]. A high cross-linking density ensures that the recognition cavities remain stable and reusable after multiple binding and washing cycles, which is especially critical in elimination-based applications such as pollutant adsorption or filtration. Commonly used cross-linkers include ethylene glycol dimethacrylate (EGDMA), divinylbenzene (DVB), and trimethylolpropane trimethacrylate (TRIM) [54,55]. While a high cross-linker-to-monomer ratio increases the mechanical and chemical resistance of the MIP, an excessively rigid structure can reduce the accessibility of binding sites and limit analyte diffusion. Thus, for applications requiring rapid binding kinetics, such as in flow-through detection sensors or dynamic pollutant remediation systems, an optimal balance must be achieved to ensure both durability and functionality [56].

A pivotal aspect of MIP performance is the template molecule, which dictates the molecular shape and chemical environment of the recognition site. In toxic molecule detection, the template is often a low-molecular-weight pollutant such as a pesticide, pharmaceutical residue, VOC, or heavy metal ion [57,58]. Direct imprinting with the target molecule is ideal for specificity but can sometimes be limited by availability, toxicity, or regulatory restrictions. In such cases, dummy templates or structurally similar analogs can be used to avoid template leakage or degradation [26,59]. For example, in the detection of methylmercury, imprinting with a safer organomercury analog can provide similar recognition characteristics while minimizing health and safety risks during synthesis. In elimination-focused applications, imprinting with environmentally persistent compounds, such as endocrine-disrupting chemicals or persistent organic pollutants (POPs), can generate MIPs suitable for long-term water treatment or solid-phase extraction processes [60].

The porogenic solvent used during polymerization significantly influences the MIP’s porosity, surface area, and the nature of monomer–template interactions [61,62]. Solvents with low dielectric constants, such as toluene or chloroform, tend to promote stronger hydrogen bonding between monomers and templates, leading to well-defined cavities [63]. In contrast, highly polar solvents like DMSO or acetonitrile may disrupt non-covalent interactions but are sometimes necessary for solubilizing polar or charged templates [51]. Importantly, the solvent also dictates the macro- and mesoporous structure of the polymer, which affects diffusion kinetics and binding site accessibility—key parameters for both detection sensitivity and adsorption efficiency. In advanced designs, mixed-solvent systems are used to tailor the microenvironment of the polymer, especially when dealing with complex templates or multifunctional monomers. Recent trends also include the use of green solvents and ionic liquids for more environmentally friendly imprinting processes, aligning with sustainability goals [25,64].

The polymerization method further defines the morphology and integration potential of MIPs [65]. Bulk polymerization, though widely used, often results in monolithic polymers that require grinding and sieving, potentially damaging recognition sites [66,67]. In contrast, precipitation polymerization and emulsion polymerization yield uniformly sized particles with higher binding site accessibility, making them ideal for sensor coatings and chromatographic applications [68,69,70]. Surface imprinting is particularly advantageous for toxic molecule detection in aqueous environments, as it enables rapid analyte access and minimal template diffusion limitations [71,72]. For elimination purposes, magnetic MIPs and core-shell nanostructures are increasingly utilized to facilitate separation and regeneration processes. Hybrid techniques involving sol–gel imprinting, electropolymerization, or layer-by-layer assembly allow precise control over polymer thickness and orientation, which is crucial for integration into microfluidic devices, portable sensors, or catalytic remediation systems [73].

Collectively, the intelligent selection and fine-tuning of these design parameters are central to the success of MIP-based platforms in both detecting and eliminating small toxic molecules. By rationally optimizing the chemical and physical attributes of the polymer network, it is possible to achieve high selectivity, sensitivity, binding kinetics, and long-term stability, even in complex real samples such as wastewater, soil, biological fluids, or food matrices. As the field continues to evolve, the integration of computational modeling, machine learning, and green chemistry principles is expected to further refine MIP design strategies, enabling next-generation sensing and purification systems that are both effective and sustainable.

## 3. Detection Strategies for Small Toxic Molecules Using MIPs

Molecularly imprinted polymers (MIPs), with their high selectivity and affinity for target analytes, are increasingly employed in sensor technologies for detecting small toxic molecules. By integrating MIPs with different signal transduction mechanisms, such as electrochemical, optical, gravimetric, or hybrid methods, researchers have developed highly sensitive and specific detection platforms. Representative strategies for small toxin detection using MIPs are summarized in Table 1. Electrochemical sensors translate binding events into electrical signals, while optical sensors rely on fluorescence or color changes. Gravimetric approaches detect minute mass variations upon analyte binding. Hybrid systems, like electrochemiluminescence, combine multiple modalities for enhanced performance. The following subsections highlight recent advances across these sensing strategies.

### 3.1. Electrochemical Methods

Electrochemical sensing transduces chemical recognition events into electrical signals, offering a powerful platform for the detection of trace-level analytes in complex matrices [93,94]. When integrated with molecularly imprinted polymers (MIPs), which provide template-specific binding sites, electrochemical sensors can achieve high selectivity alongside sensitive signal output. Typically, the binding of the target molecule to the MIP-modified electrode surface perturbs the electrochemical interface, altering parameters such as current, potential, impedance, or capacitance, thereby enabling quantitative analysis [95,96]. Common transduction techniques include cyclic voltammetry (CV), differential pulse voltammetry (DPV), square wave voltammetry (SWV), and electrochemical impedance spectroscopy (EIS).

Recent advances in MIP-based electrochemical sensing have focused on optimizing signal transduction efficiency while preserving or enhancing molecular recognition. One widely adopted approach is the in situ electropolymerization of functional monomers directly on electrode surfaces, which enables the formation of thin, uniform, and strongly adherent MIP films [97]. This technique facilitates precise control over film thickness and allows for the localization of recognition sites at the electrode interface. Han et al. [74] developed a bisphenol A (BPA) sensor by electropolymerizing p-aminobenzoic acid onto a glassy carbon electrode modified with in situ reduced gold nanoparticles. The gold nanostructures enhanced both the electron transfer rate and electroactive surface area, resulting in a detection limit of 52 nM and strong selectivity against BPA analogs such as bisphenol S and phenol. These results highlight the combined effect of imprinting fidelity and signal amplification from the nanomaterials.

**Figure 2:**
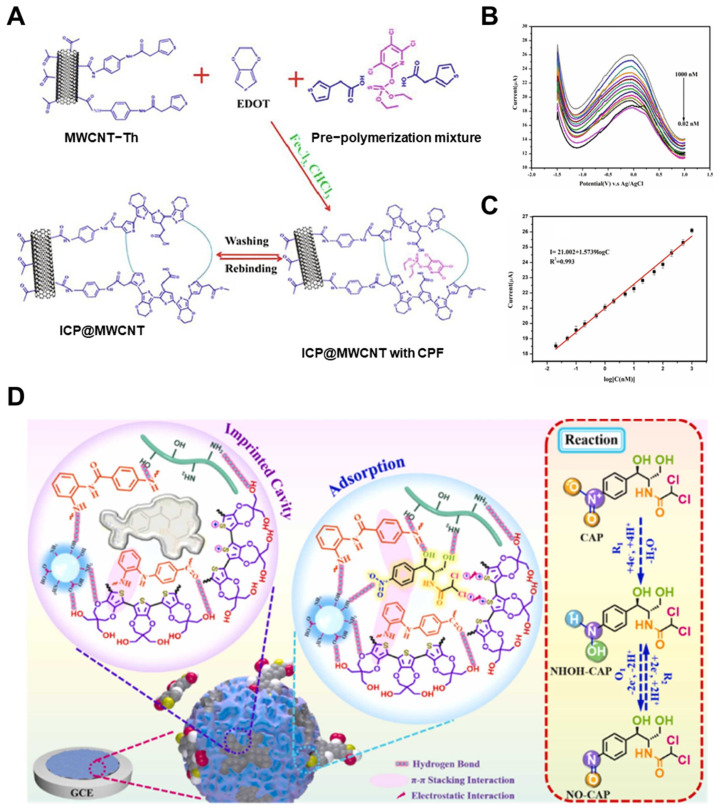
(**A**) Preparation scheme of an MIP-modified electrode based on ICP@MWCNT, (**B**) DPVs of the sensor at CPF concentrations ranging from 0.02 to 1000 nM, and (**C**) corresponding calibration curve of current response versus log[CPF] concentration (Reprinted with permission from [75]. Copyright 2022 Elsevier); (**D**) Electrochemical sensing mechanism of an MIP-based electrode (Reprinted with permission from [76]. Copyright 2022 Elsevier).

Another effective strategy involves the incorporation of conductive nanomaterials, such as multi-walled carbon nanotubes (MWCNTs), graphene oxide (GO), or mesoporous carbon, into the MIP matrix. These materials increase conductivity, expand surface area, and provide a high-loading platform for template imprinting [98,99]. In addition to carbon-based nanomaterials, polymers incorporating redox-active fullerenes have also demonstrated promising electrochemical sensing performance. Due to their unique electron-accepting properties, fullerene moieties can facilitate rapid charge transfer and enhance redox responsiveness [100,101,102]. Anirudhan et al. [75] reported a sensor for chlorpyrifos based on a copolymer of 3-thiopheneacetic acid and 3,4-ethylenedioxythiophene, electropolymerized onto an MWCNT-modified electrode (Figure 2A). The resulting MIP film achieved a detection limit of 4.0 × 10^−12^ M with a linear range from 10^−12^ to 10^−7^ M (Figure 2B,C), attributed to hydrogen bonding and π–π stacking between the template and the imprinted sites. This ultra-sensitive detection makes the system highly suitable for environmental monitoring of organophosphates. Expanding on this concept, Cheng et al. [76] designed a chloramphenicol sensor by integrating nitrogen-doped carbon derived from chitosan and a hollow polymeric substrate (PProDOT-2CH_2_OH). The MIP was synthesized via a dual-monomer system (o-phenylenediamine and p-aminobenzoic acid), forming a cross-linked network with enhanced affinity (Figure 2D). Deposited through electropolymerization onto a functionalized electrode, the resulting sensor exhibited high porosity, excellent conductivity, and a detection limit of 6.6 pM, maintaining selectivity even in the presence of structurally similar antibiotics.

Surface imprinting techniques have also gained momentum, particularly for analytes requiring rapid binding kinetics and high accessibility to recognition sites. Mani et al. [77] developed a sensor for L-tryptophan using a surface-imprinted film formed on a silanized graphene oxide substrate decorated with silver nanoparticles. The MIP layer, formed via UV-initiated polymerization of methacrylic acid and styrene, exhibited a detection limit of 3.23 × 10^−10^ M and provided excellent recovery in spiked serum samples. The silver nanoparticles facilitated electron transfer, while the GO matrix ensured high template loading and rapid response. In another study, Hu et al. [78] constructed an electrochemical sensor for zearalenone (ZEN), a mycotoxin, by depositing a MIP layer onto a hybrid electrode composed of boron-doped ordered mesoporous carbon (BOMC) and gold nanoparticles. The BOMC offered a large surface area and rapid electron transport, while gold nanoparticles enhanced both conductivity and polymerization efficiency. The resulting MIP film featured abundant accessible binding sites and achieved a detection limit of 0.1 pg/mL, with strong selectivity and stability in complex food matrices.

These examples illustrate the versatility of electrochemical MIP sensor design, where diverse fabrication strategies—from electropolymerization to nanomaterial-assisted surface imprinting—enable the development of highly selective, ultrasensitive sensors. With detection limits often reaching the picomolar or even femtomolar range, these systems are well-suited for monitoring small toxicants such as pesticides, endocrine disruptors, antibiotics, and amino acid derivatives. Moreover, the compatibility of MIPs with low-cost electrodes and scalable fabrication methods supports their integration into portable devices for real-time, on-site analysis in environmental, food, and biomedical applications.

### 3.2. Optical Methods

Optical sensing technologies detect molecular interactions by translating them into light-based signals such as fluorescence, absorbance, or refractive index changes. Unlike electrochemical methods, these techniques rely on variations in electromagnetic properties, enabling real-time, label-free, and non-invasive detection [103,104,105]. When combined with molecularly imprinted polymers (MIPs), optical sensors gain target-specific recognition through imprint cavities that modulate the local optical environment upon analyte binding. This can lead to signal shifts via mechanisms such as fluorescence quenching, energy transfer, or refractive index modulation [106,107]. The synergy between MIP selectivity and optical responsiveness makes these platforms particularly suitable for applications requiring visual readout, high sensitivity, or remote and multiplexed detection.

**Figure 3:**
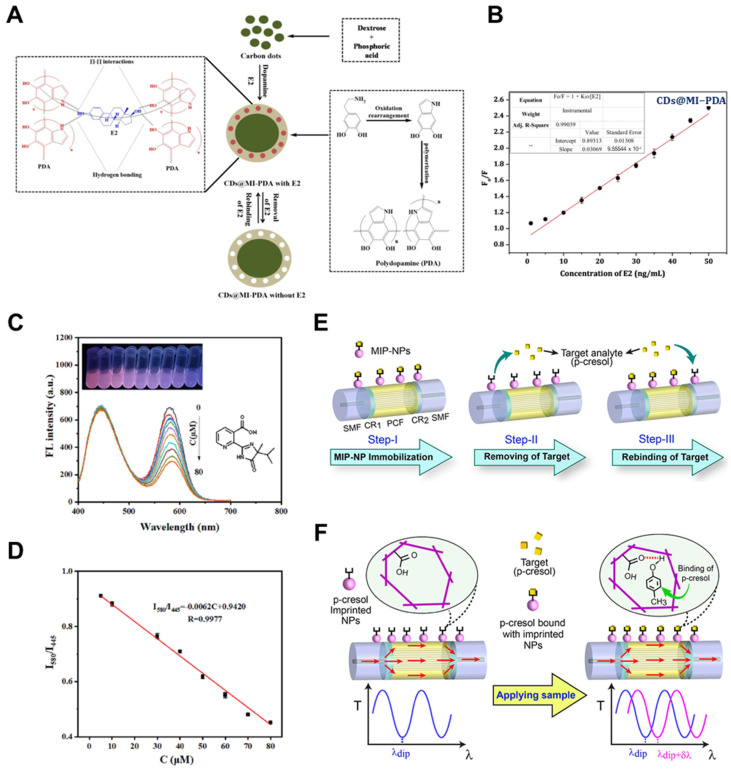
(**A**) Synthesis scheme of polydopamine-imprinted coatings over CDs (CDs@MI-PDA) for 17β-estradiol detection and (**B**) Stern–Volmer plot showing fluorescence quenching at E2 concentrations of 0–50 ng/mL (Reprinted with permission from [80]. Copyright 2022 Elsevier); (**C**) Fluorescence spectra and (**D**) calibration plots for imazapyr (IMA) detection using a molecularly imprinted ratio fluorescent (MIRF) probe (Reprinted with permission from [81]. Copyright 2024 Elsevier); (**E**) Fabrication steps and (**F**) sensing mechanism of an MIP-NP-modified SMF–PCF–SMF optical sensor for p-cresol detection (Reprinted with permission from [83]. Copyright 2023 American Chemical Society).

A prominent class of MIP-based optical sensors utilizes fluorescent nanomaterials—especially carbon quantum dots (CQDs) and semiconductor quantum dots (QDs)—as both signal reporters and imprinting scaffolds. These materials offer high quantum yields, tunable emission wavelengths, and strong environmental stability [108,109]. Wang et al. [79] developed a MIP@CQD sensor for 4-nitrophenol detection by embedding CQDs within a polymer matrix via sol–gel polymerization. Upon target binding, fluorescence quenching occurred through dynamic quenching and inner-filter effects, resulting in a detection limit of 0.41 μM and high recovery rates in real water samples. The sensor exhibited excellent photostability and dispersibility in aqueous environments. Bhogal et al. [80] further advanced this strategy by coating carbon dots with a dopamine-imprinted polydopamine layer (CDs@MI-PDA) under mild aqueous conditions. Targeting 17β-estradiol, the system relied on photo-induced electron transfer for fluorescence quenching (Figure 3A), achieving a detection limit of 0.34 ng/mL with strong selectivity in spiked biological samples (Figure 3B).

To improve signal precision and reduce environmental interference, ratiometric fluorescence sensing has gained attention [110]. Zhou et al. [81] introduced a dual-emission MIP sensor for imazapyr (IMA), combining CdTe QDs (580 nm) as the responsive probe and Si QDs (445 nm) as a stable reference. The imprinted polymer selectively quenched CdTe emission upon analyte binding, while Si QD fluorescence remained constant (Figure 3C). This design enabled a visible fluorescence shift from red to blue and allowed quantitative detection down to 1.4 μM, with high reproducibility under variable conditions (Figure 3D).

Beyond fluorescence, fiber-optic and interferometric platforms have enabled highly sensitive, label-free, real-time detection in compact formats. Liu et al. [82] constructed a long-period fiber grating (LPG) sensor functionalized with nanoMIPs synthesized via solid-phase imprinting. Targeting carboxyl-fentanyl, the binding-induced refractive index change shifted the resonance wavelength of the grating, yielding a detection limit of 50 ng/mL with excellent specificity in biological fluids. Gorai et al. [83] developed a modal interferometer based on a photonic crystal fiber (PCF) spliced between two single-mode fibers (SMFs) and coated with MIP nanoparticles for detecting p-cresol. This SMF-PCF-SMF configuration enhanced analyte–light interaction within the fiber core (Figure 3E,F), enabling sensitive detection with a limit of 1.55 nM even in untreated water samples. Both designs exemplify the potential of integrated photonics for field-deployable environmental monitoring.

Colorimetric sensors represent another class of accessible optical platforms, enabling naked-eye detection or smartphone-assisted quantification [111]. Wang et al. [84] fabricated a differential colorimetric sensor for malachite green using MIP-modified zones on a portable device equipped with a dual-path light design. A reference zone coated with non-imprinted polymers (NIPs) corrected for background signals, improving analytical accuracy. This system achieved a detection limit of 1.1 µg/L and demonstrated strong performance in on-site water testing.

Collectively, these advances highlight the breadth and adaptability of MIP-based optical sensing. Fluorescence-based sensors continue to offer ultralow detection limits and rapid response, while interferometric and colorimetric systems excel in simplicity, portability, and field applicability. Across formats, strategies such as surface imprinting, ratiometric signal design, photonic integration, and nanomaterial coupling have enabled significant improvements in sensitivity, selectivity, and matrix compatibility. Rather than converging toward a single standard, optical MIP sensors are increasingly tailored to specific applications through innovations in material chemistry and device architecture. As developments in green synthesis, miniaturization, and multiplexed signal processing continue, optical MIP technologies are rapidly maturing into robust platforms for real-time detection of small toxic molecules across environmental, food safety, and biomedical contexts.

### 3.3. Gravimetric Methods

Gravimetric sensing strategies such as quartz crystal microbalance (QCM) and surface acoustic wave (SAW) sensors have garnered increasing attention in molecularly imprinted polymer (MIP) research due to their ability to detect subtle mass variations arising from analyte binding. The integration of MIP selectivity with the real-time, label-free detection capabilities of gravimetric platforms enables sensitive and selective monitoring of small toxic molecules in both liquid and gaseous phases. Recent developments show a progressive refinement in sensor design, encompassing rational monomer selection, imprinting strategy optimization, and engineering of nanostructured transducer interfaces.

A representative early effort by Oh et al. [112] focused on surface morphology control by employing surface-initiated atom transfer radical polymerization (SI-ATRP) to fabricate BPA-imprinted poly(4-vinylpyridine-co-EGDMA) films on silica inverse opal structures. By varying polymerization time (8, 16, and 24 h), they modulated film thickness and porosity. The 24-h film exhibited the highest sensitivity (1.219 ± 0.079 Hz/nM) and selectivity (k* ≈ 2.5), while the 8-h film achieved a superior recovery rate of 94.5%. This work demonstrated how precise spatiotemporal control over polymer growth on patterned surfaces can be leveraged to optimize trade-offs between sensitivity and reversibility in QCM-based detection.

To enhance template-specific interactions at the molecular level, Aydogan et al. [85] adopted a computational design approach by modeling the coordination behavior between Cu(II) ions and the functional monomer N-methacryloyl-L-histidine methyl ester (MAH). Based on density functional theory (DFT) results, two Cu(II)-imprinted nanoparticle systems were synthesized and immobilized on QCM chips. These nanosensors (80–100 nm) achieved a low detection limit of 40.7 nM and showed excellent linearity (R^2^ = 0.99) in the range of 0.15–1.57 µM. Importantly, the Cu(II)-imprinted nanosensors exhibited strong selectivity for Cu(II) over other divalent ions such as Co^2+^, Ni^2+^, Zn^2+^, and Fe^2+^, which were tested as potential interferents in competitive binding assays. In a related approach, Liu et al. [86] utilized quantum chemical simulations to design estrone-imprinted polymers using itaconic acid (IA) as the functional monomer. The simulations identified a 1:4 template-to-monomer molar ratio as optimal, maximizing hydrogen bonding and minimizing binding energy. The synthesized MIPs, when coated on QCM electrodes, exhibited a detection limit of 16.00 µg/L and significant selectivity over structural analogs. This study exemplifies how simulation-driven monomer selection can streamline MIP formulation while enhancing recognition performance.

**Figure 4:**
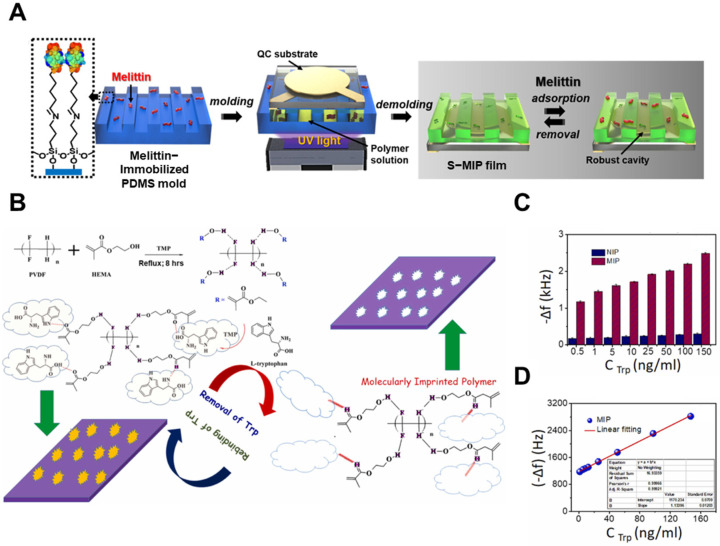
(**A**) Schematic illustration of the fabrication process for QCM sensors coated with surface-imprinted polymers (S-MIPs) (Reprinted with permission from [87]. Copyright 2023 American Chemical Society); (**B**) Synthesis scheme of a hydrophilic PVDF-based MIP, (**C**) The frequency shift (Δf) response of the MIP-SAW sensor to various tryptophan (Trp) concentrations, and (**D**) corresponding calibration curve (Reprinted with permission from [88]. Copyright 2022 Elsevier).

While the aforementioned studies focused on small molecules and ions, imprinting larger biomacromolecules remains challenging due to their size, flexibility, and structural instability. To overcome these limitations, Yang et al. [87] proposed a nanostructured surface imprinting strategy targeting melittin (MEL), a 26-residue hemolytic peptide. Using chalcone-branched polyimide (CB-PI) as a robust matrix, they employed microcontact printing and UV-induced photocrosslinking to transfer MEL-imprinted patterns onto QCM substrates (Figure 4A). The resulting MIP films exhibited a clear frequency response to MEL binding, with a response slope of 5.49 mL/mg, indicating strong analyte responsiveness. This value, reflecting the calibration curve gradient, was accompanied by excellent linearity (R^2^ = 0.999) and a low limit of detection (LOD) of 0.3 µg/mL. This approach highlighted the utility of combining chemical functionality with nanoscale patterning to achieve effective imprinting of biologically relevant peptides.

While QCM sensors are effective for liquid-phase detection, their performance may be limited in applications requiring rapid response or enhanced sensitivity to small molecules. In such cases, SAW sensors offer a promising alternative with advantages in both liquid- and gas-phase sensing. Prabakaran et al. [88] developed a SAW sensor modified with a hydrophilic poly(vinylidene fluoride) (PVDF) matrix incorporating 2-hydroxyethyl methacrylate (HEMA)-based MIPs for L-tryptophan detection. The antifouling surface was achieved via in situ polymerization within the PVDF network, balancing mechanical integrity with aqueous compatibility (Figure 4B). Upon exposure to L-tryptophan, the MIP-coated sensor exhibited a clear, concentration-dependent frequency shift, while the NIP counterpart showed minimal response (Figure 4C). As shown in Figure 4D, the calibration curve demonstrated a linear relationship across the tested range with a detection limit of 0.2 ng/mL, indicating that the integration of hydrophilic MIPs into the piezoelectric polymer platform enabled both high sensitivity and molecular selectivity. The sensor achieved a detection limit of 0.2 ng/mL and an imprinting factor of 9.34 (Figure 4C,D), while maintaining selectivity against D-tryptophan and common interferents such as ascorbic acid and tyrosine. For the detection of volatile toxicants, Wang et al. [89] synthesized silica-based MIPs targeting dimethyl methyl phosphonate (DMMP), a simulant of the nerve agent sarin. By adjusting template concentration during sol-gel synthesis, they controlled the MIP layer’s pore volume and hydroxyl density, thus influencing adsorption kinetics and sensor response. The resulting SAW sensor exhibited rapid detection of 80 ppb DMMP within 60 s and high selectivity, underscoring the effectiveness of porous silica frameworks for gas-phase MIP applications.

Collectively, these studies underscore the versatility and adaptability of MIP–gravimetric sensing platforms. From monomer-level interaction design to surface patterning and antifouling strategies, each methodological advancement contributes to the ongoing enhancement of sensitivity, selectivity, and operational robustness in QCM and SAW-based sensors. This evolution reflects a clear trajectory toward highly engineered, real-world-deployable systems for detecting a broad spectrum of toxic small molecules.

### 3.4. Hybrid Methods

While single-mode sensors based on molecularly imprinted polymers (MIPs) have achieved remarkable progress, recent advances in detection strategies have shifted toward multimodal approaches that integrate optical, electrochemical, and digital signal processing elements. Particularly, electrochemiluminescence (ECL), photoelectrochemical (PEC), and smartphone-integrated platforms have demonstrated synergistic enhancements in sensitivity, selectivity, and portability [113,114]. The evolution of these multimodal MIP-based sensors reflects a convergence of nanomaterial engineering, signal amplification techniques, and user-centric device integration.

**Figure 5:**
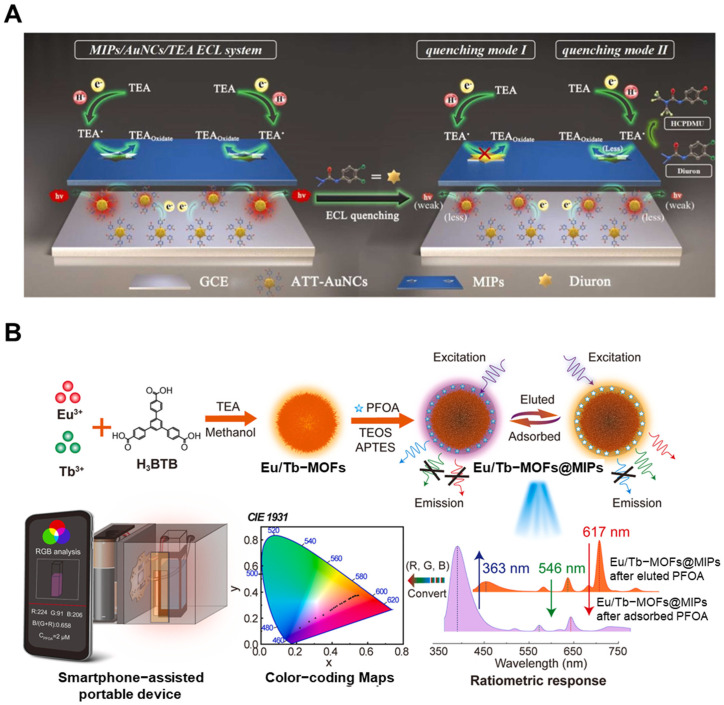
(**A**) Dual-quenching mechanisms of an MIP-ECL sensor for diuron: (I) signal blocking and (II) interaction with TEA (Reprinted with permission from [92]. Copyright 2024 Elsevier); (**B**) Preparation of Eu/Tb-MOF@MIPs and smartphone-based platform for PFOA detection (Reprinted with permission from [118]. Copyright 2024 Elsevier).

The earliest developments in this category centered on photoelectrochemical sensors. Wang et al. [90] proposed a PEC sensor for aflatoxin B1 detection based on ZnO nanorod arrays hybridized with the conducting polymer poly(3,3′-dithiophene-co-3-thiophenecarboxylic acid) (P(33DT-co-3TPCA)) and the ionic liquid 1-butyl-3-methylimidazolium chloride ([BMIM]Cl). The MIP layer provided molecular specificity, while the hybrid interface enhanced charge separation and light absorption. The sensor exhibited a linear response over 0.10–10 ng/mL and a limit of detection (LOD) of 0.058 ng/mL, highlighting the potential of PEC-MIP combinations for trace analysis under visible light. Further advancing MIP-ECL technologies, Kuang et al. [91] developed a sensor using tris(2,2′-bipyridine)ruthenium(II) complex (Ru(bpy)_3_^2+^) immobilized on ZnO–Au nanocomposites for acrylamide detection. The combination of the high surface area of ZnO and the conductivity of Au nanoparticles facilitated efficient electron transfer, while the MIP layer provided specific binding. The sensor achieved an LOD of 0.123 nM and exhibited a linear response from 1 to 108 nM, demonstrating excellent sensitivity suitable for food safety applications. In a mechanistically enhanced design, Wang et al. [92] developed a dual-quenching MIP-ECL sensor for diuron detection. The sensor was constructed by immobilizing gold nanoclusters (AuNCs), synthesized using 6-aza-2-thiothymine (ATT) as a stabilizing ligand, on a glassy carbon electrode. In the presence of triethylamine (TEA) as a coreactant, this platform leveraged two independent quenching pathways: (1) the “blocking effect,” where rebinding of diuron within MIP cavities hindered electron transfer between AuNCs and TEA• radicals, and (2) chemical consumption of TEA• by diuron through electrochemically induced hydroxylation, leading to reduced TEA• availability. This dual-mode suppression significantly enhanced signal discrimination, enabling highly sensitive and selective detection of diuron in aqueous samples (Figure 5A).

While most current MIP-based hybrid sensors rely on electrochemical and optical combinations, nonlinear optical (NLO) responses offer a promising future direction. Phenomena such as second-harmonic generation and multi-photon excitation enable amplified signal output and spatial resolution beyond conventional fluorescence. These effects can be accessed using conjugated polymers, supramolecular systems, and small chromophores [115,116]. Although MIP–NLO integration has been little explored, the high sensitivity and resolution of NLO systems suggest strong potential for future hybrid sensing strategies, especially for ultra-trace detection or multiplexed analysis.

Simultaneously, the integration of MIP systems with portable and user-friendly platforms became a prominent research focus. Zhu et al. [117] developed a smartphone-integrated optosensing platform using red-emission carbon dots (RCDs) functionalized with surface-imprinted polymers for detecting pyrethroid pesticides. Lambda-cyhalothrin, a widely used pyrethroid, was selectively captured by the RCD@SiO_2_@MIP interface. Fluorescence quenching was observed upon analyte binding, and a smartphone application converted the optical signal into quantitative readouts. The platform achieved an LOD of 0.89 µg/L via spectroscopy and 6.66 µg/L through smartphone analysis, exemplifying the integration of molecular recognition with digital detection. Building upon this paradigm, Yang et al. [118] more recently introduced a smartphone-assisted ratiometric fluorescence probe based on lanthanide metal-organic frameworks (Ln-MOFs) coated with surface MIPs for the detection of perfluorooctanoic acid (PFOA). The Eu/Tb-MOF@MIP system provided dual-emission signals (orange to purple shift) with high photostability and specificity (Figure 5B). Under UV illumination (310 nm), the fluorescence intensity ratio changed linearly with PFOA concentration (0.1–2.8 µM), with an LOD of 3.26 nM using smartphone RGB analysis. This design demonstrates how surface imprinting and multicolor ratiometric signals can enhance both accuracy and field usability.

Together, these studies reflect a clear technological trajectory: from early stage hybrid nanomaterial–based PEC and ECL systems focused on enhancing sensitivity, to dual-signal strategies that suppress background, and finally to smartphone-integrated systems emphasizing usability and real-time field detection. This evolution underscores the growing importance of not only analytical performance but also accessibility, robustness, and integration with everyday technologies in the next generation of MIP-based toxicant sensors.

While MIP-based sensing technologies continue to demonstrate impressive analytical performance, their transition into real-world applications remains constrained by key technical limitations. The reproducibility of imprinting is often hindered by uncontrolled polymer growth, leading to variability in binding site affinity and density. To address this, photoinitiated polymerization with mask-defined UV exposure or flow-through electropolymerization on microelectrode arrays can facilitate more spatially uniform and reproducible MIP films. In optical systems, conventional fluorescent probes are prone to photobleaching and low signal stability, particularly under prolonged excitation. Incorporating photostable alternatives such as nitrogen-doped carbon dots or rare-earth-doped upconversion nanoparticles offers improved durability and signal reliability. For gravimetric sensors like QCM and SAW, precise control of the MIP layer thickness (typically <100 nm) is critical for sensitivity and reproducibility, which can be achieved through surface-initiated controlled radical polymerization or spin-assisted layer-by-layer deposition. These fabrication strategies may also be adapted for high-throughput imprinting via soft lithography, enabling scalable sensor production. Therefore, to advance MIP-based sensors beyond the laboratory, it is essential to integrate precise, reproducible fabrication techniques with scalable and stable signal generation strategies.

## 4. MIP-Based Elimination Strategies for Toxic Molecules

Beyond their role in selective sensing, molecularly imprinted polymers (MIPs) have also been employed as functional materials for the active elimination of small toxic molecules. By combining molecular recognition with physical or chemical removal processes, MIPs contribute to a variety of elimination mechanisms including selective adsorption, photocatalytic degradation, and advanced oxidation. Table 2 summarizes representative approaches employing MIPs for the selective elimination of small toxic molecules. Depending on the application context, MIPs can be tailored to enhance binding affinity, improve reaction selectivity, and integrate seamlessly with catalytic or electrochemical systems [119,120,121]. The following subsections highlight recent advances in MIP-based elimination strategies, categorized by their underlying mechanisms and functional designs.

### 4.1. Selective Adsorption

Selective adsorption using molecularly imprinted polymers (MIPs) exploits the fundamental principle of molecular recognition to achieve precise removal of target contaminants from complex mixtures [134,135]. Unlike non-specific sorbents, MIPs operate by selectively pre-concentrating molecules into template-shaped cavities that match the analyte’s size, shape, and functional groups. This selectivity is especially valuable in competitive environments, such as environmental water or biological fluids, where structurally similar compounds coexist. In MIP-based adsorption systems, the recognition event is not just a passive interaction but actively governs uptake, enabling faster adsorption kinetics, improved binding affinity, and minimized interference [136]. When engineered on porous, high-surface-area substrates or combined with responsive materials (e.g., magnetic or photocatalytic components), these systems can also exhibit enhanced mass transfer, recyclability, and integration with downstream processes [137,138,139].

One prominent approach leverages MIP-coated membranes for simultaneous filtration and adsorption. Wang et al. [122] engineered a PVDF membrane functionalized with a dual-layer MIP coating for selective removal of dimethomorph. Using a polydopamine intermediate and surface polymerization, they ensured tight integration of recognition sites onto the membrane interface. The MIP membrane demonstrated high uptake capacity (314 µg/cm^2^), robust selectivity against analogs, and exceptional reusability over five adsorption–desorption cycles. Notably, in ginseng extracts—a highly complex matrix—the membrane selectively removed the fungicide while preserving valuable plant constituents, showcasing its potential for food-grade purification.

Another effective design incorporates ionic liquids and noble metal nanostructures for fast, selective antibiotic capture. Chen et al. [123] prepared an ionic-liquid-based MIP layer on gold–carbon nanospheres (Au-CNS) via surface-initiated ATRP for oxytetracycline (OTC) adsorption. The use of ionic liquid as both functional monomer and crosslinker enhanced polarity compatibility with OTC, while the high conductivity and surface area of Au-CNS accelerated equilibrium (within 15 min). The MIP exhibited an adsorption capacity of 56.7 mg/g and retained performance over multiple reuse cycles. This illustrates how rational monomer–template interactions, paired with nanostructured supports, can fine-tune adsorption strength and kinetics.

**Figure 6:**
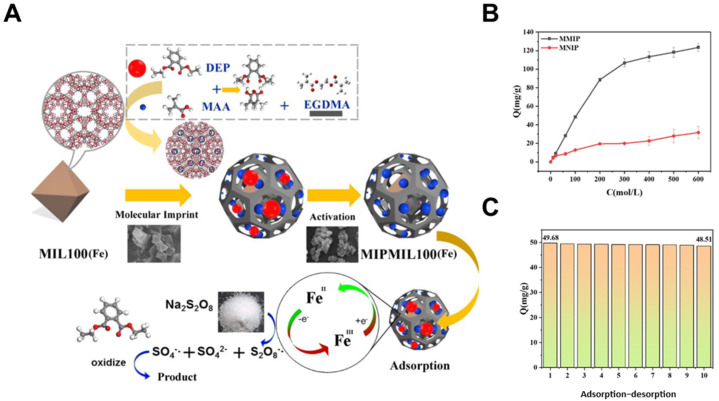
(**A**) Schematic of MIP formation via hydrogen-bonded copolymerization of DEP and MAA within MOF channels, followed by imprinting and template removal to create selective binding sites (Reprinted with permission from [124]. Copyright 2020 Elsevier); (**B**) Adsorption isotherms curves of cyproheptadine (CYP) on MMIPs and MNIPs, and (**C**) regeneration performance of MMIPs. (Reprinted with permission from [125]. Copyright 2021 Elsevier).

Incorporating MIPs into metal-organic frameworks (MOFs) allows for additional synergy between adsorption capacity and selective recognition [140,141]. Li et al. [124] constructed a surface-imprinted MIL-100(Fe) composite targeting diethyl phthalate (DEP), using in situ polymerization to distribute recognition sites both inside and outside the MOF pores (Figure 6A). The MIP showed a fourfold increase in binding compared to the non-imprinted material, reaching 13.6 mg/g. Remarkably, the system also functioned as a catalytic platform for persulfate-activated degradation, demonstrating how selective adsorption can serve as a pre-concentration step in multifunctional treatment schemes.

Magnetically retrievable MIPs further enable practical reuse without filtration steps. Cao et al. [125] synthesized Fe_3_O_4_-core magnetic MIPs for selective elimination of tricyclic antidepressants like cyproheptadine. The imprinted layer provided shape and site-specific interactions, yielding an adsorption capacity of 134.9 mg/g and maintaining efficiency after 10 cycles (Figure 6B,C). High recoveries in real river water (94–106%) confirmed the MIP’s environmental compatibility and specificity under field-like conditions.

Collectively, these studies show how selective adsorption via MIPs can be tailored through surface chemistry, imprinting architecture, and substrate selection. By optimizing the physicochemical affinity toward target molecules while minimizing non-specific binding, MIP-based adsorbents serve as robust, regenerable platforms for pollutant-specific removal in environmental and food systems.

### 4.2. Photocatalytic Degradation

Photocatalytic degradation offers a powerful route for the mineralization of organic pollutants under ambient conditions, but its practical application has long been hindered by a lack of molecular selectivity [142,143,144]. Conventional photocatalysts, such as TiO_2_ or CeO_2_, typically degrade pollutants in a non-specific manner, often reacting preferentially with abundant, low-toxicity coexisting species rather than rare but highly toxic targets [145,146]. To overcome this, molecular imprinting has been combined with photocatalysis to introduce selective recognition. In these systems, target molecules are first specifically bound within imprinted cavities on the photocatalyst surface, where localized degradation occurs upon light exposure. This site-specific reaction enhances selectivity over non-specific, solution-based degradation.

The feasibility of introducing molecular selectivity through imprinting was first validated in simple oxide systems. Fiorenza et al. [147] synthesized molecularly imprinted TiO_2_ via a sol–gel approach using 2,4-dichlorophenoxyacetic acid (2,4-D) and imidacloprid as templates, achieving 60% degradation of 2,4-D under UV light within 120 min, compared to only 30% by the non-imprinted control. Their work demonstrated that even without polymer matrices, imprinting alone could endow a photocatalyst with target-specific degradation capability. To increase efficiency and responsiveness under visible light, structural modifications such as heterojunction formation were introduced. Zhang et al. [126] developed a TiO_2_@Fe_2_O_3_@g-C_3_N_4_ composite incorporating a double Z-scheme pathway and MIP layers targeting sulfamethoxazole (SMX). This system achieved 96.8% degradation of SMX under visible light, while exhibiting minimal degradation of analogues like sulfadiazine and bisphenol A. The work highlighted that coupling molecular recognition with efficient charge separation can simultaneously improve selectivity and photocatalytic performance.

Further refinement was achieved through surface engineering to improve imprint quality and accessibility. Zhang et al. [127] utilized single-crystalline TiO_2_ nanorods with exposed (111) facets as a platform for imprinting dimethyl phthalate (DMP). The imprinted photoanode achieved 92.1% DMP removal within 60 min, outperforming the 58.3% efficiency of non-imprinted TiO_2_ even under a tenfold excess of natural organic matter. These results emphasized the value of crystallographic control in optimizing the spatial fidelity of recognition sites. Expanding on this foundation, porous architectures were integrated to further enhance surface area and radical activation efficiency. Ding et al. [148] introduced a MIP-functionalized Fe-MOF-74@SiO_2_ photocatalyst for persulfate-activated degradation of DMP. The system achieved over 90% degradation within 30 min (26 mM PS, 0.02 g/L catalyst), while retaining high selectivity in the presence of chloride ions. This design showcased how imprinting not only guides adsorption but also steers radical reactivity toward the template molecule even under chemically challenging conditions.

**Figure 7:**
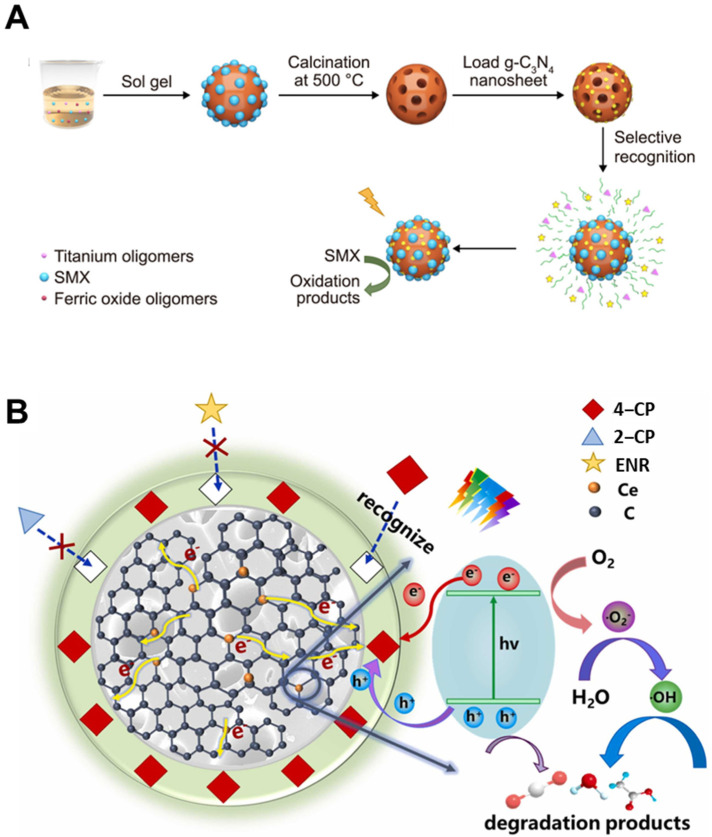
(**A**) Fabrication process of molecularly imprinted TiO_2_@Fe_2_O_3_@g-C_3_N_4_ (MFTC) photocatalyst for selective degradation of sulfamethoxazole (SMX) (reprinted with permission from [126]. Copyright 2024 Elsevier); (**B**) mechanism of selective adsorption and photodegradation of 4-chlorophenol (4-CP) using MIP-CeO_2_@BC photocatalyst (reprinted with permission from [128]. Copyright 2025 Elsevier).

With these architectural and functional advances established, research expanded toward real-world deployment. Li et al. [149] addressed practical applications by constructing a green-synthesized g-C_3_N_4_@Mn–FeOOH@MIP photocatalyst targeting nine heterocyclic amines (HCAs), known carcinogens in processed foods (Figure 7A). The system achieved >80% HCA degradation in coffee samples, maintained >70% recovery, and showed >85% reusability over five cycles. This study demonstrated the translational potential of MIP-based photocatalysts for food safety and consumer protection under realistic conditions. In parallel, environmental robustness was explored to ensure functionality in natural water systems. Lyu et al. [128] synthesized a CeO_2_@biochar photocatalyst imprinted for p-chlorophenol (4-CP) (Figure 7B), which achieved 94% removal under visible light and retained >60% efficiency in the presence of humic acid, glucose, or trypsin. The incorporation of biochar served not only as a stable support but also as an electron conduit, enhancing charge mobility and enabling consistent performance even in highly variable aqueous matrices.

Together, these studies outline a coherent evolution of MIP-based photocatalytic technology—from basic proof-of-concept systems to structurally optimized catalysts and finally to practical applications in real and complex environments. The persistent role of molecular imprinting in enabling molecular-level specificity underscores its centrality in advancing next-generation photocatalysts. Looking forward, integration of MIPs with stimuli-responsive materials, multi-target recognition systems, or continuous-flow reactors may open new frontiers in precision water purification and environmental sensing.

### 4.3. Advanced Oxidation Activation

Advanced oxidation processes (AOPs) have proven to be powerful tools for degrading recalcitrant organic pollutants, owing to their ability to generate reactive oxygen species (ROS) such as hydroxyl radicals (•OH), sulfate radicals (SO_4_•^−^), and singlet oxygen (^1^O_2_) [150,151,152]. However, the inherent non-selectivity of these radicals, along with their short lifetimes (typically <40 μs for SO_4_•^−^ and <20 ns for •OH), limits their effectiveness in complex aqueous environments and at low pollutant concentrations [153,154]. The integration of molecularly imprinted polymers (MIPs) with AOP catalysts has emerged as an effective solution, enabling selective preconcentration of target pollutants at catalytic interfaces and guiding the generated radicals toward specific degradation pathways.

**Figure 8:**
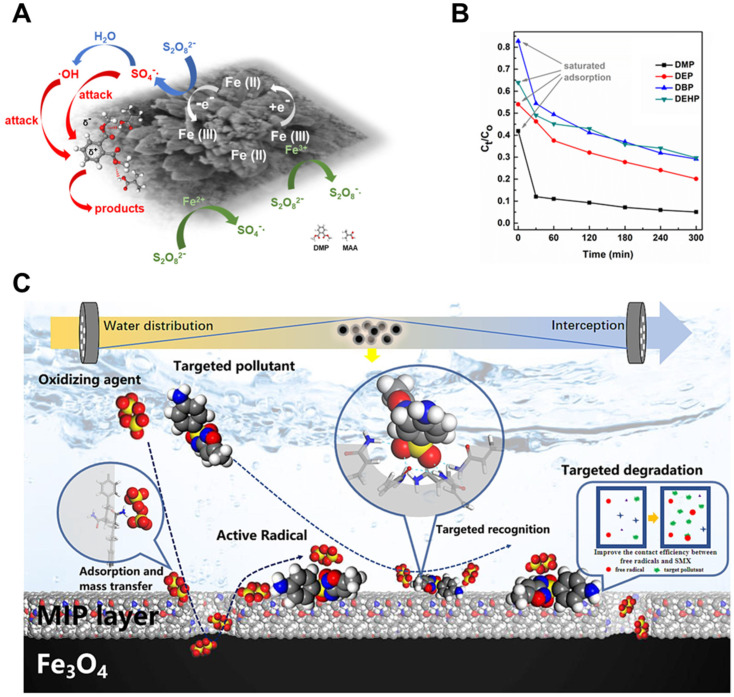
(**A**) Proposed activation mechanism of persulfate (PS) by Fe-MOF-74/MIP and (**B**) its selective performance toward dimethyl phthalate (DMP) (reprinted with permission from [130]. Copyright 2021 Elsevier); (**C**) schematic of targeted pollutant degradation in a molecularly imprinted catalytic membrane reactor (MICMR) (reprinted with permission from [133]. Copyright 2023 Elsevier).

A representative example of this strategy was demonstrated by Ding et al. [130], who constructed a molecularly imprinted Fe-MOF-74 catalyst (Fe-MOF-74/MIP) for the targeted degradation of dimethyl phthalate (DMP) using persulfate (PS) as the oxidant (Figure 8A). The MIP layer selectively enriched DMP near the Fe catalytic centers through hydrogen bonding and electrostatic interactions, thereby enhancing the local concentration of the pollutant at the reactive interface. This synergistic effect led to a 1.5-fold increase in the catalytic degradation rate, achieving nearly 90% DMP removal within 30 min (Figure 8B). Their results emphasized that the imprinting layer did not merely serve as an adsorbent but actively facilitated oxidation by optimizing the spatial proximity between DMP and generated radicals. Similarly, Xie et al. [131] synthesized a surface-imprinted NH_2_-MIL-53(Fe) catalyst (MIP-AA) tailored for sulfamethoxazole (SMX) degradation. The imprinted sites enabled specific pre-adsorption of SMX via weak interactions such as hydrogen bonding, enriching the pollutant around catalytic centers. Notably, radical quenching experiments identified •OH as the dominant reactive species, while the MIP layer did not impede radical generation. Rather, it facilitated pollutant proximity, reducing degradation byproducts and simplifying pathways.

Shifting focus to metal-free systems, Tang et al. [132] proposed an innovative approach by developing a molecularly imprinted polydopamine (MI-PDA) catalyst that could directly in situ activate peroxydisulfate (PDS) via its electron-deficient nitrogen sites, producing singlet oxygen (^1^O_2_) as the main oxidative species. Unlike conventional systems relying on metal active sites, the MI-PDA exploited electron-deficient nitrogen sites to cleave the S–O bond in PDS, directly generating singlet oxygen (^1^O_2_) for SMX degradation. This dual functionality of selective enrichment and radical-free activation yielded over 95% removal efficiency and significantly enhanced reaction kinetics, representing a green and efficient strategy for selective oxidation. To further amplify both adsorption and catalytic performance, Yi et al. [155] fabricated a Fe-MOFs@MIP hybrid catalyst, combining the catalytic activity of Fe-MOFs with a dopamine-based imprinting layer tailored for SMX. The polydopamine (PDA) coating enhanced not only target molecule recognition through its functional –NH_2_ and –OH groups but also electron transfer dynamics, which boosted ROS generation. The resulting system achieved over fivefold enhancement in degradation rate, supported by density functional theory (DFT) and EPR studies that identified active species and degradation pathways.

Lastly, to address issues of catalyst recovery and practical deployment, Li et al. [133] designed a molecularly imprinted catalytic membrane reactor (MICM) by integrating MIP-functionalized Fe_3_O_4_ nanoparticles into a PVDF membrane. This composite not only facilitated targeted pollutant capture but also adsorbed oxidants such as PMS, H_2_O_2_, and PS, enabling both free radical (e.g., •OH, SO_4_•^−^) and non-radical (^1^O_2_) reaction pathways (Figure 8C). In PMS systems, the MICM achieved over 90% removal of SMX within 150 min, and kinetic analysis showed the reaction rate constants increased up to 33-fold compared to the bare Fe_3_O_4_ catalyst. The membrane structure provided a confined reaction microenvironment, enhancing the mass transfer efficiency of both oxidants and pollutants.

Collectively, these studies underscore the pivotal role of molecular imprinting in enhancing advanced oxidation activation. Whether through site-specific adsorption, radical generation near target molecules, or alternative non-radical pathways, MIP-integrated catalysts represent a robust platform for selective and efficient degradation of small toxic molecules in complex aqueous environments.

### 4.4. Coupled Systems and Multifunctional Platforms

While single-function molecularly imprinted polymers (MIPs) have demonstrated selective recognition and removal of target pollutants, complex environmental matrices often demand more versatile systems. To address this, researchers have increasingly engineered MIP-based platforms that couple multiple functional components or integrate into structured devices, enhancing selectivity, catalytic efficiency, and environmental adaptability.

**Figure 9:**
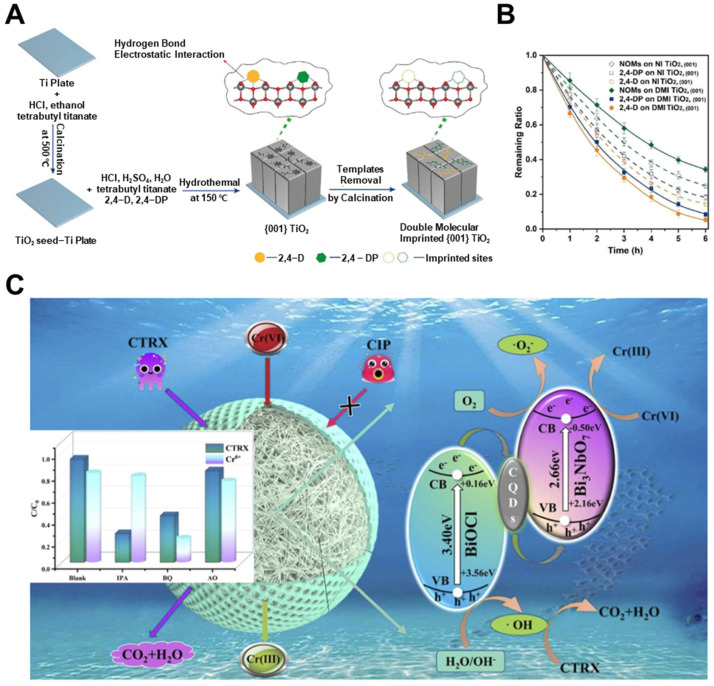
(**A**) Preparation of DMI TiO_2_ (001) electrode and (**B**) degradation profiles of 2,4-D, 2,4-DP, and natural organic matters using non-imprinted (NI) and DMI TiO_2_ (001) electrodes (reprinted with permission from [156]. Copyright 2022 Elsevier); (**C**) mechanism of selective redox removal of ceftriaxone (CTRX) and Cr(VI) using a bifunctional MIP-based photocatalyst (reprinted with permission from [129]. Copyright 2024 Elsevier).

A notable example of dual-functionality was introduced by Tang et al. [156], who fabricated a double molecularly imprinted (DMI) TiO_2_ photoelectrode with shape-selective cavities for both 2,4-dichlorophenoxyacetic acid (2,4-D) and 2,4-dichlorophenoxypropionic acid (2,4-DP) (Figure 9A). Constructed on a crystalline (001) TiO_2_ surface, the electrode exhibited highly selective photocatalytic degradation, removing over 90% of both herbicides in ternary mixtures while minimizing interference from natural organic matter (Figure 9B). This dual-template approach demonstrated how multifunctional recognition can be implemented on structurally defined catalysts to handle co-existing, structurally similar pollutants in complex matrices.

Moving from oxidative to reductive mechanisms, Lou et al. [157] developed an oxygen-vacancy engineered molecularly imprinted TiO_2_ cathode (MI-TiO2–x) for the electroreductive dehalogenation of florfenicol (FLO). The material combined surface imprinting for selective adsorption and oxygen vacancies for accelerated charge transfer, enabling efficient direct electron transfer. The system achieved a rate constant of 0.021 min^−1^, surpassing many state-of-the-art electrocatalysts, and crucially, suppressed the propagation of antibiotic resistance genes (ARGs) in real swine wastewater—an important step toward ecological risk mitigation.

Recognizing the need for simultaneous multi-pollutant removal and material reusability, Hu et al. [158] developed a dual-template magnetic MIP (CBZ/OXC-MIPs) targeting carbamazepine (CBZ) and oxcarbazepine (OXC). Functional monomers were optimized through molecular simulations, and the resulting porous, magnetic polymer exhibited fast equilibrium (30 min), high specific affinity, and robust recyclability, with only a 3.39% loss in capacity after 10 reuse cycles. This system showcased how integrating magnetic functionality and dual-template imprinting can lead to scalable and regenerable MIP adsorbents for pharmaceutical contaminants. The concept of multifunctionality was further extended by Zhang et al. [159], who constructed a carbon quantum dot (CQDs)-doped, molecularly imprinted heterojunction photocatalyst (CQDs-MIP-BNO) capable of synchronous redox removal of both ceftriaxone sodium (CTRX) and hexavalent chromium [Cr(VI)]. The CQDs enhanced charge separation in the Z-scheme BiOCl/Bi_3_NbO_7_ heterojunction, while MIP layers enabled high target specificity. The system achieved 94% CTRX and 80% Cr(VI) removal within 45 min and was successfully implemented in a practical flow-through photocatalytic reactor, validating its real-world applicability.

Lastly, Zhang et al. [129] applied a surface molecularly imprinted activated carbon (MIP@AC) within a simulated permeable reactive barrier (PRB) for the in situ remediation of naphthalene (NAP) in groundwater (Figure 9C). When coupled with peroxymonosulfate (PMS), the MIP@AC achieved adsorption and degradation selectivity coefficients of 31.47 and 10.88, respectively, over non-imprinted controls. The imprinted cavity served as a spatially confined reaction site, effectively reducing ROS self-quenching and enabling targeted oxidation under natural groundwater conditions.

Collectively, these studies illustrate a clear trajectory in MIP system design—from dual recognition capabilities to catalytic site engineering to stimuli-responsive or separable hybrid systems. By coupling MIPs with photo-, electro-, and magnetic functionalities and embedding them into operational devices, researchers have opened new pathways toward selective, robust, and field-deployable solutions for small toxic molecule elimination in challenging environments.

Although MIP-based systems show great promise for the selective removal of toxicants, they often face operational limitations under realistic environmental or biological conditions. A major issue is the reduction in adsorption efficiency after repeated use, which results from irreversible fouling or mechanical instability of the polymer matrix. One effective solution is to graft antifouling polymer brushes—such as poly(ethylene glycol) or zwitterionic chains—onto the MIP surface to minimize nonspecific adsorption and maintain site accessibility. For magnetic MIPs commonly used in batch processes, employing a core–shell structure (e.g., Fe_3_O_4_@SiO_2_@MIP) improves mechanical rigidity and supports more complete template removal. In flow-through remediation, incorporating MIP particles into membrane or packed-bed reactors requires control over particle dispersion and porosity; this can be optimized by using templated porous silica supports or porogenic solvent systems during synthesis. Finally, to enable sustainable reuse, standardized regeneration protocols using mild solvents (e.g., ethanol or diluted acid) under optimized pH conditions are needed to maintain binding site fidelity. Therefore, improving structural robustness, antifouling properties, and regeneration strategies is essential to realize MIP-based systems as reliable, reusable platforms for toxicant removal.

## 5. Conclusion and Future Perspectives

The utilization of molecularly imprinted polymers (MIPs) in the detection and elimination of small toxic molecules has proven to be a powerful and adaptable approach. Owing to their high selectivity, sensitivity, and versatility, MIPs are increasingly used in critical areas such as environmental monitoring, food safety, and biomedical diagnostics. Through the combination of various transduction methods, such as electrochemical, optical, gravimetric, and hybrid sensing systems, MIPs enable the development of highly effective sensors capable of detecting trace analytes in complex matrices.

Recent advancements in MIP-based detection strategies have highlighted the significant potential of these materials. Electrochemical sensors have demonstrated excellent performance in identifying toxicants such as pesticides, endocrine disruptors, and mycotoxins, owing to their high sensitivity and specificity. Optical sensors, particularly those incorporating nanomaterials like carbon quantum dots and semiconductor quantum dots, achieved ultra-low detection limits and strong signal stability, making them ideal for real-time and label-free analysis. Gravimetric sensors, including quartz crystal microbalance and surface acoustic wave devices, have also provided label-free, real-time monitoring of small toxic molecules, further demonstrating the versatility of MIPs in various settings. Hybrid sensing platforms that combine multiple detection modalities have shown enhanced performance, making MIP-based systems more robust and adaptable to field-based applications.

In addition to detection, MIPs also show significant promise for the active removal of toxic molecules. Techniques such as MIP-coated membranes, magnetic MIPs, and MIP-integrated photocatalytic or electrochemical systems have been effectively applied for environmental remediation, offering sustainable strategies for decontaminating complex samples. Despite these advances, certain challenges persist, notably reproducibility of MIP synthesis, potential matrix interference in complex samples, and the need for further miniaturization and cost reduction. Addressing these obstacles will require the development of more sophisticated polymer matrices with improved molecular recognition capabilities, along with optimized sensor designs for practical applications.

To further elevate the performance of MIP-based platforms, future research should focus on refining polymer synthesis and recognition accuracy. Challenges such as non-uniform binding sites and limited reusability continue to hinder widespread application. Emerging techniques like microfluidic-assisted imprinting, 3D-printed or modular MIP scaffolds, and stimuli-responsive materials offer promising routes for more reproducible and scalable synthesis. Additionally, computational modeling tools including molecular dynamics and quantum chemical simulations can accelerate rational design by optimizing monomer–template interactions, reducing experimental trial-and-error. Meanwhile, ongoing innovations in MIP chemistry, such as novel functional monomers and nanomaterial integration, are further improving recognition efficiency, chemical and thermal stability, and overall performance under demanding environmental and biological conditions. Simultaneously, advancements in microfabrication and sensor miniaturization are driving the development of MIP-based sensors to be more affordable and scalable, facilitating their integration into portable and wearable platforms. The convergence of MIPs with smartphone-compatible readers and Internet of Things (IoT) technologies may enable real-time, autonomous monitoring with minimal user intervention. Finally, the integration of artificial intelligence (AI) and machine learning (ML) into MIP-based systems may transform these platforms into intelligent devices capable of advanced recognition with predictive modeling and real-time data interpretation.

In conclusion, the convergence of advanced materials science, intelligent computation, and sustainable engineering is positioning MIP-based technologies as next-generation solutions for toxicant detection and environmental remediation. As the field continues to mature, MIPs are expected to play a pivotal role in tackling global challenges in environmental sustainability, food safety, and public health. With their unique combination of high specificity, adaptability, and integration with portable and smart platforms, MIP-based systems are poised to become essential components of future intelligent and sustainable monitoring infrastructures.

## Figures and Tables

**Table 1:** Representative MIP-based strategies for small toxin detection.

Detection Method	Templates	Imprinting Technique	Polymerization Methods	MIP Format	Linear Range	LOD	Real Sample	Recovery/%	Ref.
Electrochemical Sensing	Bisphenol A	NR	Electropolymerization	Film	0.5–100 μmol/L	52 nmol/L	Tap water, milk, orange juice, bottle	96.7–107.6	[74]
	Chlorpyrifos	Surface imprinting	in-situ polymerization	Film	0.02–1000 nmol/L	4.0 nmol/L	Tap water and cucumber	NR	[75]
	Chloramphenicol	NR	Electro polymerization	Film	0.0001–125 μmol/L	6.6 pmol/L	Eye drops, honey, tap water, aquaculture wastewater	99.2–102.8	[76]
	L-Tryptophan	Surface imprinting	NR	Film	NR	3.23 × 10^−10^ mol/L	Blood	98.0–102.0	[77]
	Zearalenone	Dummy template imprinting	Electro polymerization	Film	5 × 10^−4^–1 ng/mL	1 × 10^−4^ ng/mL	Corn, rice, beer	98.6–112.9	[78]
Optical Sensing	P-nitrophenol	Sol-gel imprinting	NR	Nanoparticle	0–144 μmol/L	0.41 μmol/L	Tap water, wastewater, seawater	95.1–107.8	[79]
	17β-oestradiol	Surface imprinting	Oxidative polymerization	Nanoparticle	1–50 ng/mL	0.34 ng/mL	Tap and river water	96.4–102.2	[80]
	Imazapyr	Sol-gel imprinting	NR	Nanoparticle	5.0–80.0 μmol/L	1.4 μmol/L	Soil and puerariae lobatae radix	85.5–98.0	[81]
	Butyrylfentanyl	Solid-phase imprinting	NR	Nanoparticle	0–1000 ng/mL	50 ng/mL	Human serum	NR	[82]
	p-cresol	NR	Thermal polymerization	Nanoparticle	1.55 × 10^−9^–10^−3^ mol/L	1.55 nmol/L	Tap water	93–105	[83]
	Malachite green	Surface imprinting	Thermal polymerization	Nanoparticle	0–60 μg/L	1.1 μg/L	Water	94.1–105	[84]
Gravimetric Sensing	Cu(II) ions	Surface imprinting	Emulsion polymerization	Nanoparticle	0.15–1.57 μmol/L	40.7 nmol/L	Water	NR	[85]
	Estrone	Surface imprinting	NR	Nanoparticle	NR	16.00 μg/L	NR	NR	[86]
	Melittin	Microcontact imprinting	Photo polymerization	Film	1–30 μg/mL	0.3 μg/mL	NR	100.3–107.9	[87]
	L-Tryptophan	Surface imprinting	in-situ polymerization	Film	0.5–150 ng/mL	0.2 ng/mL	NR	NR	[88]
	Dimethyl methyl phosphonate	NR	Hydrolysis polymerization	Nanoparticle	NR	80 ppb	NR	NR	[89]
Photoelectrochemical Sensing	Aflatoxin B1	Surface imprinting	Electrochemical polymerization	Film	0.10–10 ng/mL	0.058 ng/mL	Water	93.5–112.8	[90]
	Acrylamide	Sol-gel imprinting	Electro polymerization	Film	1–10^8^ nmol/L	0.123 nmol/L	Potato chips and cookies	99.8–104.7 93.3–102.3	[91]
	Diuron	NR	Electro polymerization	Nanoparticle	NR	2.16 × 10^−12^ g/mL	Water	94.6–103	[92]

NR—Not reported.

**Table 2:** Representative MIP-based strategies for small toxin elimination.

Elimination Method	Templates	Imprinting Technique	Polymerization Method	Adsorption Capacity	Removal Efficiency	Ref.
Selective Adsorption	Dimethomorph	Surface imprinting	Oxidative polymerization	314 μg/cm^−2^	95.67%	[122]
	Oxytetracycline	Surface imprinting	ATRP	56.7 mg/g	NR	[123]
	Diethyl phthalate	Surface imprinting	NR	13.6 mg/g	NR	[124]
	Tricyclic analogues	Surface imprinting	Bulk polymerization	123.5 mg/g	NR	[125]
Photocatalytic Degradation	Sulfamethoxazole	NR	EISA method	20 mg/g	96.8%	[126]
	Phthalic acid esters	Surface imprinting	Thermal polymerization	NR	91.3%	[127]
	p-chlorophenol	Surface imprinting	Thermal polymerization	32.3 μg/L	94%	[128]
	Ceftriaxone Sodium Cr(VI)	Surface imprinting	NR	NR	94% and 80%	[129]
Advanced Oxidation	Dimethyl phthalate	NR	Bulk Polymerization	NR	90%	[130]
	Sulfamethoxazole	Surface imprinting	NR	38.04 mg/g	NR	[131]
	Sulfamethoxazole	Surface imprinting	Self-assembly	11.04 mg/g	95%	[132]
	Sulfamethoxazole	Surface imprinting	NR	948.1 mg/L	90%	[133]

ATRP—Atom transfer radical polymerization; EISA—Evaporation-induced self-assembly; NR—Not reported.

## Data Availability

Not applicable.
